# Bean Leaf Beetle (*Ootheca* spp.) (Coleoptera: Chrysomelidae) Management via Planting Timing and Insecticides

**DOI:** 10.3390/insects13080709

**Published:** 2022-08-07

**Authors:** Charles Halerimana, Samuel Kyamanywa, Samuel Olaboro, Pamela Paparu, Stanley T. Nkalubo, John Colvin, Robert A. Cheke, Darren J. Kriticos, Michael H. Otim

**Affiliations:** 1 Department of Agricultural Production, College of Agricultural and Environmental Sciences, Makerere University, Kampala P.O. Box 7062, Uganda; 2 National Coffee Research Institute, Kituza, Mukono P.O. Box 185, Uganda; 3 National Crops Resources Research Institute, Namulonge, Kampala P.O. Box 7084, Uganda; 4 Natural Resources Institute, University of Greenwich at Medway, Central Avenue, Chatham Maritime, Kent ME4 4TB, UK; 5 Commonwealth Scientific and Industrial Research Organization, GPO 1700, Canberra 2601, Australia; 6 Cervantes Agritech, 7 Plummer St., Weetangera 2614, Australia

**Keywords:** bean leaf beetles, common bean, *Ootheca* spp., planting time, spray regimes, foliar damage

## Abstract

**Simple Summary:**

Bean leaf beetles (*Ootheca* spp.) (Coleoptera: Chrysomelidae) are a major pest of the common bean (*Phaseolus vulgaris* L.) in Africa, attacking the roots, leaves, floral parts, and young pods, and reducing grain yields significantly. However, there is no comprehensive program for managing bean leaf beetles. In Uganda, farmers typically try to reduce pest impacts by delaying bean crop sowing, and to a lesser extent, using insecticides, but there is no information on the impact of delayed planting and insecticide application on bean leaf beetles. We conducted a study to assess the effects of planting timing and insecticide spray regimes on bean leaf beetle abundance, foliar damage, and common bean grain yield in three agro-ecological zones. Bean leaf beetle abundance was highest in mid-planting compared to late and early planting, while foliar damage was highest in late planting in two of the three agro-ecological zones. However, marketable grain yield was highest in early-planted plots in all three agro-ecological zones. Insecticide application reduced foliar damage and increased marketable grain yield, with a combination of soil drench and foliar spray resulting in significantly less foliar damage and higher grain yield. Marketable grain yield was higher when insecticides were combined with early planting in all agro-ecological zones.

**Abstract:**

Bean leaf beetles (*Ootheca* spp.) (Coleoptera: Chrysomelidae) are one of Africa’s most important pests of the common bean (*Phaseolus vulgaris* L.). Roots, leaves, floral parts, and young pods are all attacked, leading to a considerable loss in grain yield. In Uganda, there are no comprehensive prescribed management strategies for bean leaf beetles, but farmers typically try to control the pest by delaying bean crop sowing, and to a lesser extent, using insecticides. Although farmers have consistently implemented the two approaches, there is no information on the effects of the approaches in Uganda. To assess the impact of planting timing and insecticide spray regimes on bean leaf beetle populations, concomitant foliar damage, and grain yield, we set up trials in three agro-ecological zones with known presence of the beetles during the second rainy season of 2016 (2016) and the first rainy season of 2017 (2017). The first planting, coinciding with early planting, was conducted within one week after the onset of rains. The second planting, coinciding with mid planting, followed two weeks later, while the third planting, considered late planting in this study, was conducted one month after the second planting. A foliar application of cypermethrin commencing at 7 days after emergence (DAE), 14 DAE, 21 DAE, 28 DAE, and 35 DAE; a soil drench of imidacloprid at planting combined with a foliar spray starting at 7 DAE; and an untreated control were among the insecticide spray regimes evaluated. Higher bean leaf beetle abundance was recorded from mid-planting, while higher foliar damage was recorded from late planting in two of the three agro-ecological zones. However, higher marketable grain yield was recorded from early planting in all agro-ecological zones, suggesting that delayed planting may not be beneficial. Insecticide application reduced foliar damage and increased marketable grain yield, with a combination of soil drench and foliar spray resulting in much less foliar damage and, as a result, higher grain yield. However, this did not result in economic benefits. Furthermore, marketable grain yield was higher when insecticide spray regimes were combined with early planting in all agro-ecological zones during both seasons. Our findings suggest that the common bean should be planted early and that the control of the bean leaf beetle should target both the adults and the juvenile stages in the soil. Therefore, there is a need for farmers to be able to access less-expensive soil treatments.

## 1. Introduction

Bean leaf beetles (*Ootheca* spp.) (Coleoptera: Chrysomelidae) are a major pest of the common bean (*Phaseolus vulgaris* L.) in Africa, attacking the roots, leaves, floral parts, and young pods, and thereby reducing grain yields significantly [1]. Beans are an important component of the diets of most Africans, and threats to bean production directly impact food security, especially for subsistence households [2]. However, despite being identified as significant pests of beans in Africa more than a quarter of a century ago, relatively little is known about these beetles and their management.

*Ootheca* species are native to Africa and are mainly found in tropical Africa and Madagascar [1], with a total of 13 species reported to date [3]. The most common species are *Ootheca mutabilis* (Sahlberg) and *O. bennigseni* (Weise) [4]. *Ootheca mutabilis* is found in most regions of continental Africa, from Senegal to southern Sudan in the north to Angola and north-eastern South Africa in the south, while *O. bennigseni* is found in central, eastern, and southern Africa [3,4]. *Ootheca mutabilis*, *O. proteus*, and *O. orientalis* are the three *Ootheca* species found in Uganda, with *O. mutabilis* being the most widely dispersed [5,6]. Distinguishing these species in the field is difficult, and previous research has likely suffered from some taxonomic confusion. The species display various color combinations and are distinguished based on the differences in the male genitalia, which are not conclusive for females [3,4].

*Ootheca* species have a wide host range, mostly including food legumes such as cowpea [*Vigna unguiculata* (L.) Walp.], Bambara groundnut [*Vigna subterranean* (L.) Verdc.], pigeonpea [*Cajanus cajan* (L.) Millsp.], soybean [*Glycine max* (L.) Merr.], hyacinth bean [*Lablab purpureus* (L.) Sweet.], and common bean (*P. vulgaris*) [3,4]. The pest also feeds on cucumber (*Cucumis sativus* L.), roselle (*Hibiscus sabdariffa* L.) [3], and other leguminous trees, specifically Sesbania [*Sesbania sesban* (L.) Merr.] and *Crotolaria grahamiana* Wight. and Arn [7].

Adult beetles attack common bean seedlings, making irregular and circular holes in the leaves and occasionally destroying the crop [8,9]. Larvae feed on lateral roots and nitrogen-fixing nodules, resulting in regions of the field with yellowed, stunted plants that may dry up prematurely or produce empty pods [8,9,10,11]. *Ootheca mutabilis* causes similar damage to cowpeas as it does to beans, but it also spreads viral diseases including cowpea yellow mosaic virus and the cowpea strain of tobacco mosaic virus [12]. There is no available information on the grain yield losses due to *Ootheca* species in Uganda. Elsewhere, *O. mutabilis* alone was found to induce yield losses ranging from 19.5 to 59.8% in cowpeas in Nigeria [12]. In Tanzania, foliar feeding caused by *O. bennigseni* alone was estimated to result in bean yield reductions of 18 to 31% [13]. 

The bean crop grown in Uganda is a key domestic food and income source for the country’s rural population [14]. However, yields are relatively low, with an average of 1.5 t ha^−1^ [15], compared to up to 2.5 t ha^−1^ for bush varieties and 3.5 t ha^−1^ for climbing bean kinds [16]. Bean yield is low due to abiotic and biotic factors [2,8], which include insect pests such as *Ootheca* spp. [10].

Given the paucity of information on *Ootheca* spp., it is necessary to consider perspectives drawn from ecological and biological analogues such as *C. trifurcata*. Manipulation of planting time is one of the most useful tactics for reducing the impacts of *C. trifurcata* [17,18,19] and has been applied successfully to many pest problems and crops [18,20,21,22,23,24]. In the United States, planting of soybean (*G. max*) near the end of the recommended planting period reduced early colonization and subsequent pod injury by *C. trifurcata* [17,18]. In Nigeria, varying the sowing date of cowpea (*V. unguiculata*) from 2 July to 18 August reduced populations of *Maruca vitrata* [22]. On the contrary, early and late planting of pigeon pea (*C. cajan*) in April and October, respectively, resulted in higher infestation of *Helicoperva armigera* compared to mid-planting in July in Nigeria [22]. Furthermore, while delayed planting reduced infestation by *M. vitrata* and pod-feeding bugs (*Clavigralla tomentosicollis* and *Anoplocnemis curvipes*) in north-eastern Nigeria, this was not consistent with thrips (*Megalurothrips sjostedti*) [23]. It has been suggested that there is usually a build-up of pests as the season progresses, which results in increased damage to late-planted crops [23]. Furthermore, early planting enables the crop to escape high temperatures during the flowering stages when the crop is sensitive to heat [23]. Farmers in northern Uganda delay bean sowing in order to avoid damage by bean leaf beetles, especially during the long rainy season [10], but it is unknown whether farmers’ practice of delaying common bean planting results in less foliar damage due to bean leaf beetles and, as a result, higher yields. 

The use of insecticides is the principal recourse for the control of insect pests in cultivated food legumes [22]. In cowpeas (*V. unguiculata*), application of insecticides lowered pest densities and improved yields [25,26,27,28]. In French beans (*P. vulgaris*), application of insecticides reduced thrips infestation and increased yield [29,30,31]. In soybeans (*G. max*), application of insecticides reduced the abundance of *C. trifurcata* and subsequent leaf loss but did not result in yield increase [20].

There are no officially recommended measures by pest management specialists against bean leaf beetles in Uganda. Nonetheless, some farmers try to avoid the pest impacts by applying insecticides. Application of insecticides is unnecessary if there is no effect on grain yield by insect pests [20], but there is scarcity of information on the effect of insecticides on bean leaf beetle populations, associated foliar damage, and grain yield in Uganda. The purpose of this study, therefore, was to determine the effect of planting time and insecticide spray regimes on bean leaf beetle abundance, associated foliar damage, and grain yield in Uganda. Specifically, the objectives of this study were: (i) to determine the appropriate planting date that would reduce bean leaf beetle attacks and increase grain yield of common bean in Uganda and (ii) to determine the most economical insecticide spray regime for use in the management of bean leaf beetles in Uganda.

## 2. Materials and Methods

### 2.1. Study Sites

**Figure 1:**
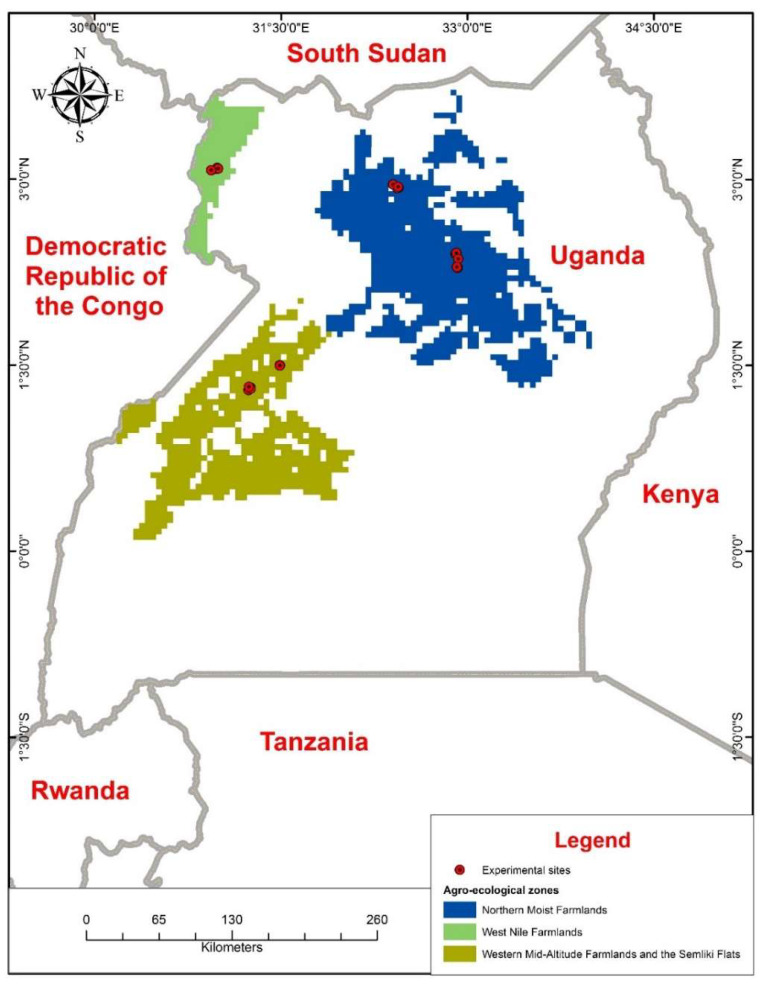
Map of Uganda showing the locations of experimental sites in different agro-ecological zones. Arc GIS (ESRI, Redlands, CA, USA) was used to generate the Map.

The study was conducted during the short rainy season (September–November) of 2016 and the long rainy season (March–June) of 2017, hereafter referred to as 2016 and 2017, respectively, in three agro-ecological zones: Northern Moist Farmlands, West Nile Farmlands, and Western Mid-Altitude Farmlands and Semliki Flats (Figure 1), hereafter referred to as NMF, WNF, and WMAFSF, respectively. These three agro-ecological zones, according to [5], are among Uganda’s most bean leaf beetle infested places. Rainfall (amount and distribution), temperatures, relative humidity, soil properties (organic matter, PH, available P, and exchangeable Ca and K), terrain, agricultural systems, and land use types differ between these zones [32]. Table S1 shows the average amounts of rainfall, temperatures, and relative humidity in the three agro-ecological zones over the study period. In each agro-ecological zone, the district where the Zonal Agriculture Research and Development Institute (ZARDI) is located was selected. In NMF, Lira district, where Ngetha ZARDI is located, was selected. In WNF, Arua district, where Abi ZARDI is located, was selected. In WMAFSF, Hoima district, where Bulindi ZARDI is located, was selected. In each district, trials were established at the ZARDI, hereafter referred to as on station, and in farmer-owned fields, hereafter referred to as on farm. However, on-farm studies in NMF during 2017 were established in Gulu district because of the vast size of this agro-ecological zone and high infestation in the district. Fields held by farmers were chosen depending on the crops grown the previous season. Fields with a crop that was a host for bean leaf beetles were chosen, as well as villages where bean leaf beetles had been a severe problem in the past.

### 2.2. Treatments and Experimental Layout

Sowing occurred at three different times in each agro-ecological zone. Early planting was conducted within one week after the onset of rains. Mid planting was conducted two weeks after early planting, while late planting was conducted one month after mid planting. Table 1 shows the different sowing dates for each planting time in different agro-ecological zones. At each planting date, an experiment was established on farm and on station except for Lira and Gulu districts, where the planting date on farm (in Gulu district) was not the same as on station (in Lira district) during 2017. It should also be noted that the onset of rains is not always at the same time in the three agro-ecological zones.

For each planting, six spray regimes together with an untreated control were applied. The spray regimes included:

1. Spray regime 1: Six foliar applications of cypermethrin (Cyper lacer 5% E.C) at a rate of 0.075 kg a.i. ha^−1^, each applied weekly starting 7 days after the emergence (DAE) of the bean crop to 42 DAE.
2. Spray regime 2: Five foliar applications of cypermethrin (Cyper lacer 5% E.C) at a rate of 0.075 kg a.i. ha^−1^, each applied weekly starting from 14 DAE to 42 DAE.
3. Spray regime 3: Four foliar applications of cypermethrin (Cyper lacer 5% E.C) at a rate of 0.075 kg a.i. ha^−1^, each applied weekly starting from 21 DAE to 42 DAE.
4. Spray regime 4: Three foliar applications of cypermethrin (Cyper lacer 5% E.C) at a rate of 0.075 kg a.i. ha^−1^, each applied weekly starting from 28 DAE to 42 DAE.
5. Spray regime 5: Two foliar applications of cypermethrin (Cyper lacer 5% E.C) at a rate of 0.075 kg a.i. ha^−1^, each applied weekly starting from 35 DAE to 42 DAE.
6. Spray regime 6: Soil drenching with Imidacloprid (200G/L Imidacloprid) at a rate of 2.1 kg a.i. ha^−1^ at planting combined with a weekly foliar spray of cypermethrin (Cyper lacer 5% E.C) at a rate of 0.075 kg a.i. ha^−1^ starting from 7 DAE to 42 DAE.
7. Control: Plots that were not treated with insecticide served as a control.

Insecticide treatments were applied in a randomized complete block design with four replicates. Each treatment plot measured 5 m by 3 m. Two bean seeds of Kawula, a local farmer’s favorite variety, were planted in each hole at a 50 cm by 20 cm hole spacing. Experimental plots were surrounded with five rows of the same variety as buffer rows. No fertilizer was applied to any plots at any sites in this experiment. Plots were weeded twice during the growing period, first at 4 weeks after planting and second at 8 weeks after planting. Spraying regimes were varied in order to generate distinct populations of adults and foliar damage. To suppress bean leaf beetle larvae in the soil, spray regime 6 included a soil drench. However, data on larval abundance and damage were not collected, as they would require destructive sampling. A Cooper Pegler CP knapsack sprayer (Cooper Pegler, Paris, lle-de-France, France) with a 15 L capacity was used for foliar spraying. A complete cone nozzle with a flow rate of 0.5 L min^−1^ was used on the sprayer. A watering can was used for soil drenching. To ensure consistency of the drench, the distance between the rosette of the watering can and the surface was maintained at 30 cm in all plots that received the drench. All spraying was completed at 42 DAE, when the bean crop was podding and bean leaf beetles were thought to be less damaging.

### 2.3. Sampling for Bean Leaf Beetle Abundance and Foliar Damage

From 7 DAE to 42 DAE, data on the occurrence and severity of foliar damage were collected weekly. *Ootheca* species are most active between 9 and 11 a.m. [5]; therefore, sampling was conducted then. A total of ten plants randomly selected from each of the plot’s three center rows were sampled. Data on bean leaf beetle populations were collected through direct counting, and foliar damage was scored using a visual scale of 0 to 5, where 0 = no defoliation, 1 = 1–5% defoliation, 2 = 6–25% defoliation, 3 = 26–50% defoliation, 4 = 51–75% defoliation, and 5 = 76–100% defoliation [13]. Each plot’s beans were harvested, threshed, and divided into marketable and non-marketable grains before being weighed. A moisture meter (MiniGAC 2500, Dickey-John Corporation, Auburn, IL, USA) was used to determine the moisture level of the grain at harvest, and the weight of the grain was adjusted to 14 percent moisture content. This was accomplished by applying the following formula: adjusted weight = [100-moisture content measured at harvest/100-base moisture of 14%] × measured weight at harvest [33]. Each plot’s yield was then standardized to kg ha^−1^.

### 2.4. Data Analysis

The data were first subjected to a mixed design analysis of variance model. Season, agro-ecological zone, planting time, and spray regimes were evaluated as fixed effects while site (on farm and on station) and replicates were treated as random effects. Bean leaf beetle counts were transformed into square root (x + 0.5) before statistical analysis to homogenize variances. Normal distribution and homogeneity of variance were tested using the Shapiro–Wilk test in R. From the model, differences between the means for significant interactions were tested using contrast post hoc tests with lme4 and multicomp packages. Differences were considered significant at α = 0.05. Furthermore, regression analysis was used to determine the relationship between foliar damage and marketable grain yield. All the analyses were performed using R statistical program version 4.1.2 [34].

### 2.5. Profitability of Spray Regimes

The profitability of spray regimes was determined using a cost–benefit analysis. The total cost of each spray regime was a sum of the cost of insecticide and its application. To determine the cost of spray regimes, the amount of spray mixture used on each experimental plot was recorded at every time of application. The corresponding amount of insecticide used per plot was calculated and used to generate the amount of insecticide and hence the cost for 1 ha. Furthermore, the cost of each time of application was USD $14.6 for foliar sprays, which includes labor costs and the knapsack sprayer. Cost of application of spray regime six includes a watering can, which cost USD $4.4. Marketable grain yield per plot was also standardized to kg ha^-1^ and multiplied by the price of 1 kg of dry beans to determine gross returns. Net returns were also calculated by subtracting the total costs (costs of insecticide and application costs) from the gross returns. As used in [31], marginal rate of return was used to determine whether each spray regime was profitable. Marginal rate of return is the ratio of net returns to costs of each spray regime. If the marginal rate of return was larger than one, a spray regime was considered profitable. The farm gate price of 1 kg of common bean was USD $0.73 at the time of the experiment, with US $1 equaling 3420 Uganda shillings. 1 L of cypermethrin cost $5.9 dollars, while 1 L of imidacloprid cost $35.1 dollars.

## 3. Results

### 3.1. Bean Leaf Beetle Abundance

There were significant (*p* < 0.001) effects of season, agro-ecological zone, planting time, and spray regimes on bean leaf beetle abundance (Table S2). There were also significant (*p* < 0.001) effects of season × agro-ecological zone, season × planting time, agro-ecological zone × planting time, and season × agro-ecological zone × planting time (Table S2). However, there were no significant (*p* > 0.05) effects of season × spray regimes, agro-ecological zone × spray regimes, season × agro-ecological zone × spray regimes, season × planting time × spray regimes, and season × agro-ecological zone × planting time × spray regimes (Table S2). 

There was significantly (df = 4, F = 7.3, *p* < 0.001) higher bean leaf beetle abundance in mid planting than early and late planting in WNF during 2016 and both NMF and WNF during 2017. However, there were no significant differences in bean leaf beetle abundance across planting times in NMF during 2016 or WMAFSF during either season (Table 2). 

**Figure 2:**
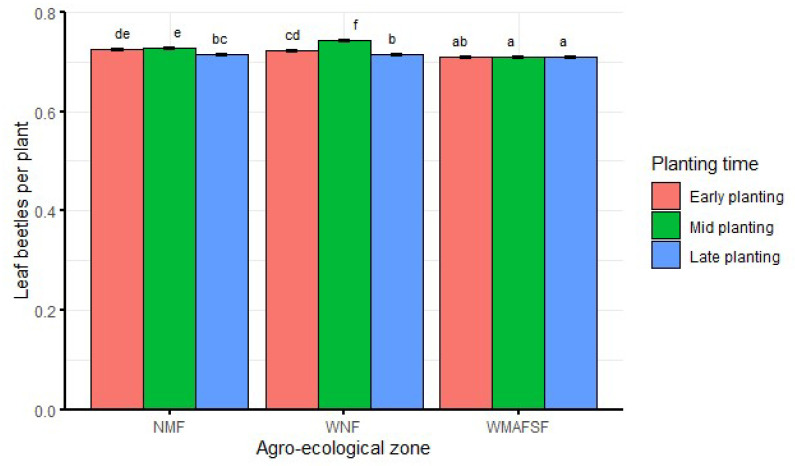
Interaction effects of agro-ecological zone and planting time on bean leaf beetle abundance averaged over seasons. Bars bearing the same letters represent means (±SE) that are not significantly different at α = 0.05.

When averaged over seasons, significantly (df = 4, F = 31.8, *p* < 0.001) higher bean leaf beetle abundance was recorded during mid planting than both early and late planting in WNF (Figure 2). There were no significant differences between early and mid-planting in NMF; however, the two plantings recorded significantly higher bean leaf beetle abundance than late planting. There was no significant difference in bean leaf beetle abundance between planting times in the WMAFSF (Figure 2).

**Figure 3:**
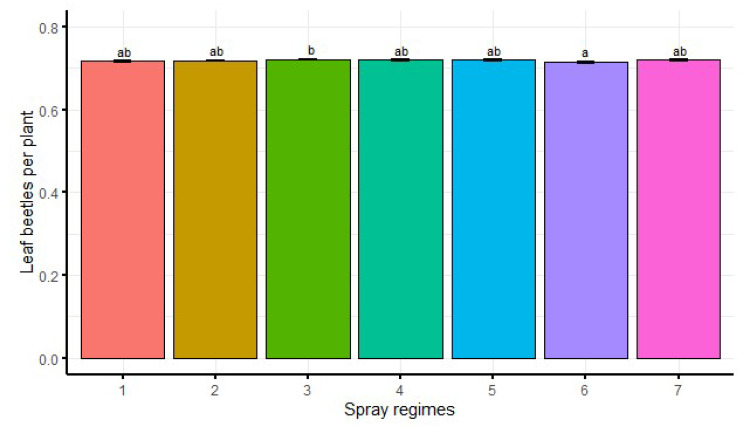
Main treatment effects of spray regimes on bean leaf beetle abundance. Bars bearing the same letters represent means (±SE) that are not significantly different at α = 0.05.

The main treatment effects of spray regimes were significant (df = 6, F = 2.7, *p* < 0.05) on bean leaf beetle abundance (Figure 3). Significantly lower bean leaf beetle abundance was recorded from spray regime 6 than spray regime 3. There were no significant differences between the other spray regimes (Figure 3).

### 3.2. Foliar Damage

All main effects, two-factor, three-factor, and four-factor interactions were significant (*p* < 0.001) for foliar damage (Table S3). However, the four-factor and selected three-factor interactions are not presented due to complexity of the interactions. Factors which are not presented in three-way interactions are presented in two-way interactions.

There was significantly (df = 4, F = 18.1, *p* < 0.001) higher foliar damage in mid planting than early and late planting in WNF during both seasons. However, there was higher foliar damage in late planting than early and mid-planting in WMAFSF during both seasons and in NMF during 2017. There was no significant difference in foliar damage between mid and late planting in NMF during 2016 (Table 3). 

There was significantly (df = 12, F = 7.6, *p* < 0.001) lower foliar damage from spray regime 6 in all agro-ecological zones in both seasons (Table 4). In addition, foliar damage generally increased from spray regime 1 to spray regime 5 in all agro-ecological zones during both seasons. Furthermore, the control had significantly higher foliar damage than all the spray regimes in all agro-ecological zones during both seasons (Table 4).

There was significantly (df = 12, F = 3.0, *p* < 0.001) lower foliar damage from spray regime 6 across the three planting times in both seasons. However, spray regime 6 was not significantly different from spray regime 1 in 2017. Contrarily, there was significantly higher foliar damage from the control across planting times and seasons (Table 5). Generally, foliar damage increased from spray regime 1 to spray regime 5 in all planting times in both seasons (Table 5).

**Figure 4:**
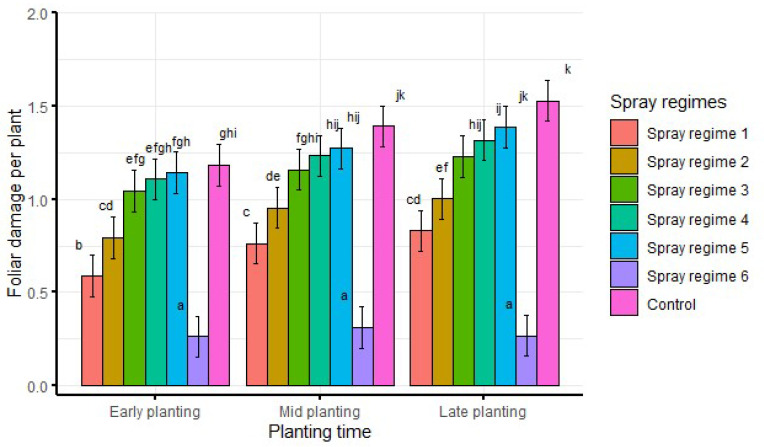
Interaction effects of planting time and spray regimes on foliar damage averaged over seasons and agro-ecological zones. Bars bearing the same letters represent means (±SE) that are not significantly different at α = 0.05.

When averaged over seasons, there was significantly (df = 12, F = 2.5, *p* < 0.05) lower foliar damage from spray regime 6 across all planting times. Similarly, there was significantly higher foliar damage from the control than other spray regimes across all planting times (Figure 4). Generally, foliar damage increased from spray regime 1 to spray regime 5 across all the planting times (Figure 4).

**Figure 5:**
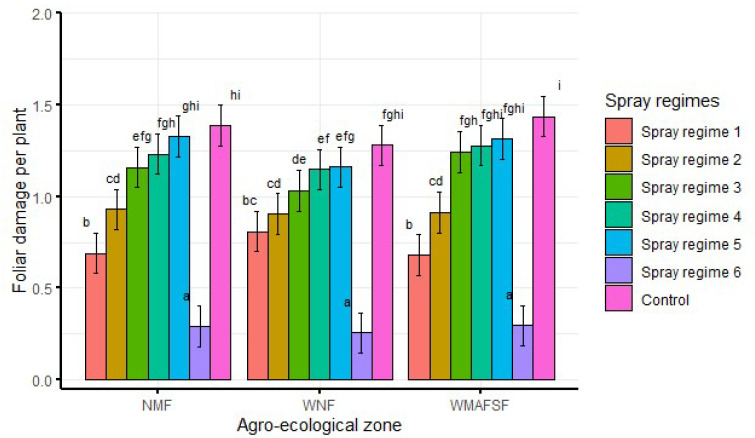
Interaction effects of agro-ecological zones and spray regimes on foliar damage averaged over seasons and planting times. Bars bearing the same letters represent means (±SE) that are not significantly different at α = 0.05.

When averaged over planting times and seasons, significantly (df = 12, F = 3.5, *p* < 0.001) lower foliar damage was recorded from spray regime 6 in all agro-ecological zones. Additionally, highest foliar damage was recorded from the control across all agro-ecological zones (Figure 5). Generally, foliar damage increased from spray regime 1 to spray regime 5 in all agro-ecological zones. However, foliar damage was only significantly higher in control than spray regimes 6, 1, 2 and 3 but was not different in the control and spray regimes 4 and 5 (Figure 5).

**Figure 6:**
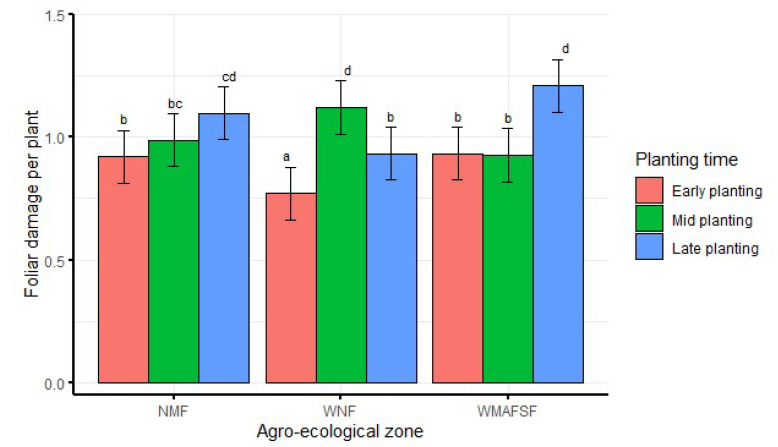
Interaction effects between agro-ecological zone and planting time on foliar damage averaged over seasons. Bars bearing the same letters represent means (±SE) that are not significantly different at α = 0.05.

When averaged over seasons, significantly (df = 4, F = 24.6, *p* < 0.001) higher foliar damage was recorded from mid-planting in WNF and from late planting in WMAFSF (Figure 6). There was no significant difference between early and mid-planting in NMF and WMAFSF. Furthermore, there was no significant difference between mid and late planting in NMF (Figure 6).

### 3.3. Marketable Grain Yield

All main effects, two-factor, three-factor, and four-factor interactions were significant (*p* < 0.001) for marketable grain yield (Table S4). However, the four-factor and selected three-factor interactions are not presented due to complexity of the interactions. Factors which are not presented in three-way interactions are presented in two-way interactions

There was significantly (df = 4, F = 111.6, *p* < 0.001) higher grain yield from early planting than both mid and late planting in all agro-ecological zones during 2016. Similarly, there was higher marketable grain yield during early planting in WNF during 2017. However, there was higher marketable grain yield during late planting than mid and early planting in NMF and WMAFSF during 2017. Furthermore, there was higher marketable grain yield during mid planting than late planting in WNF during 2017 (Table 6).

Significantly (df = 12, F = 2.3, *p* < 0.05) higher marketable grain yield was recorded from spray regime six in all agro-ecological zones in both seasons (Table 7). Generally, there was a reduction in marketable grain yield from spray regime 1 to spray regime 5 in all agro-ecological zones in both seasons. The control had significantly lower marketable grain yield compared to spray regime 6 but was not necessarily significantly different from other spray regimes (Table 7).

Spray regime 6 had significantly (df = 12, F = 5.5, *p* < 0.001) higher grain yield than other spray regimes across all planting times in both seasons (Table 8). Similarly, the control had the lowest marketable grain yield across all planting times in both seasons. Generally, there was a marketable grain yield reduction from spray regime 1 to spray regime 5 across all planting times in both seasons. The control had significantly lower marketable grain yield compared to spray regime 6 but was not necessarily significantly different from other spray regimes (Table 8).

**Figure 7:**
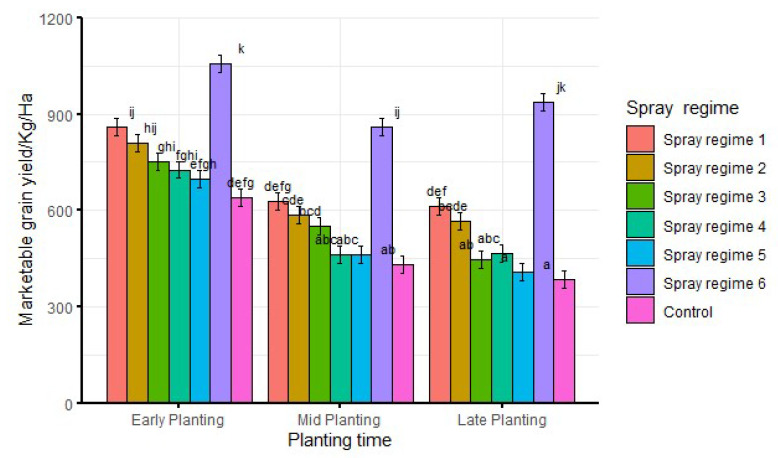
Interaction effects of planting time and spray regimes on marketable grain yield averaged over season and agro-ecological zone. Bars bearing the same letters represent means (±SE) that are not significantly different at α = 0.05.

When averaged over season and agro-ecological zone, there was significantly (df = 12, F = 1.9, *p* < 0.05) higher marketable grain yield from spray regime 6 than other spray regimes across all planting times (Figure 7). The control had significantly lower marketable grain yield compared to spray regime 6 but was not necessarily significantly different from other spray regimes. Furthermore, significantly higher marketable grain yield was recorded from all spray regimes during early planting than corresponding spray regimes in both mid and late planting (Figure 7).

**Figure 8:**
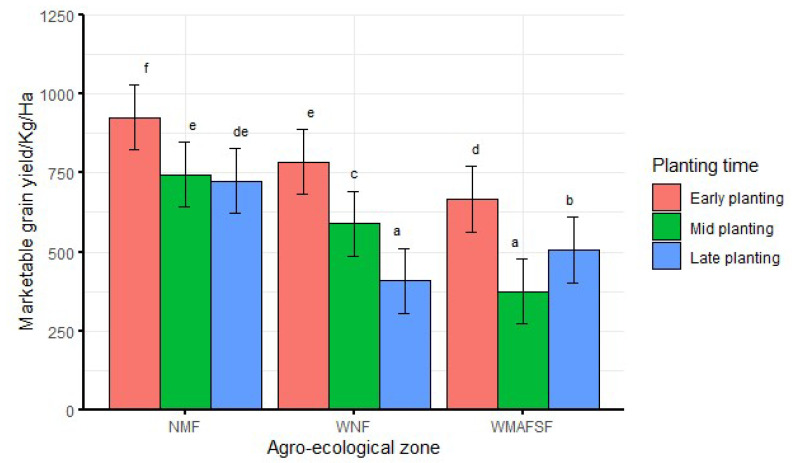
Interaction effects of agro-ecological zone and planting time on marketable grain yield averaged over seasons. Bars bearing the same letters represent means (±SE) that are not significantly different at α = 0.05.

When averaged over season, significantly (df = 4, F = 24.1, *p* < 0.001) higher marketable grain yield was recorded from early planting in all agro-ecological zones (Figure 8). Late planting had the lowest marketable grain yield in NMF and the WNF. There was no significant difference between mid and late planting in NMF. Across agro-ecological zones, the lowest marketable grain yield was recorded during mid planting in WMAFSF but was not significantly different from late planting in WNF (Figure 8). 

### 3.4. Relationship between Foliar Damage and Marketable Grain Yield

There was a negative relationship between foliar damage and marketable grain yield at all planting times, sites, agro-ecological zones, and seasons except for mid planting on farm in WNF during 2016 (Table 9). Furthermore, the negative relationship was significant (*p* < 0.05) across all planting times, sites, agro-ecological zones, and seasons except for early planting on farm in NMF during both seasons, early planting on station in NMF during 2017, and mid planting on station in WNF during 2017 (Table 9). Additionally, the exception of positive relationship in mid planting on farm in WNF during 2016 was not significant (*p* > 0.05).

### 3.5. Profitability of Different Spray Regimes

The marginal rate of return was greater than one for spray regime 2, 3, 4, and 5, and less than one in spray regime 1 and 6 in NMF (Table S5). Only spray regime 5 resulted in a marginal rate of return that is greater than 1 in both WNF and WMAFSF. There was a negative marginal rate of return associated with spray regime 6 in all three agro-ecological zones (Table S5).

## 4. Discussion

Our findings indicate that higher bean leaf beetle abundance was recorded from 2017 than that from 2016 in both NMF and WNF. Similarly, there was higher foliar damage during 2017 than 2016 in NMF, but the opposite was true in WNF and WMAFSF. In addition, grain yield was higher in 2017 than 2016 in NMF and WNF but not in WMAFSF. Differences in weather conditions during 2016 and 2017 in the three agro-ecological zones may be responsible for the differences in bean leaf beetle abundance, foliar damage, and marketable grain yield. Weather conditions affect pest densities and damage [35], but also affect grain yield output [36,37]. There was higher rain fall, temperatures, and humidity during 2017 than in 2016 in the NMF (Table S1). There was higher rainfall and humidity during 2016 than 2017 in WNF and WMAFSF, but temperatures were higher during 2017 than 2016. The interacting effects of the weather conditions may be responsible for the differences in bean leaf beetle abundance, foliar damage, and yield between seasons. Future studies on modelling the effect of weather conditions on bean leaf beetles will be important in ascertaining this. Additionally, future studies should involve the two seasons more than once to determine if the trends would be the same. 

Our findings also indicate that the effect of planting time on bean leaf beetle abundance and foliar damage was not consistent across agro-ecological zones. There was a higher bean leaf beetle abundance during the mid-planting in NMF and WNF, while there was a higher abundance during the early planting in WMAFSF. Higher numbers of insect pests in early planting have been reported [20]. This is because overwintering insect pests are attracted to the earliest emerging plants [20]. *Ootheca* species are reported to undergo obligatory diapause and the emergence of the teneral adults is influenced by crop emergence and the onset of rain [1,8]. Probably, emergence of teneral adults from diapause is responsible for higher bean leaf beetle abundance in early planting. However, larger insect populations in late planting have also been reported [18,21,24,38]. Migration of insects from early- to late-planted crops is one of the causes of pest build up in late-planted crops [18]. This could be the cause of high bean leaf beetle abundance in late-planted beans, but information is lacking on the migratory behavior of bean leaf beetles. High insect populations in late planting have also been reported in insects that complete more than one generation in a season [21]. In early planting, there is early crop maturity before the occurrence of the second generation [21]. While it is not clear how many generations bean leaf beetles complete in a season, their development takes between 60–250 days depending on temperature and relative humidity [1]. Recent studies on the developmental biology of *Ootheca* species in Uganda indicate that their development takes approximately 120 days [39], suggesting that high populations in beans planted 6 weeks after the emergence of teneral adults could be due to the second generation. There was higher foliar damage in during mid planting in WNF, but foliar damage was higher during late planting in NMF and WMAFSF. Higher foliar damage during mid planting in WNF can be attributed to the high bean leaf beetle abundance. However, the common bean is associated with various defoliators, including the larvae of butterflies and moths, that could be responsible for the high foliar damage that cannot be explained by low leaf beetle populations in WMAFSF during late planting. 

There was higher marketable grain yield in early planting than mid planting, which was in turn higher than late planting except in WMAFSF, where only late planting had higher marketable yield than mid planting. Higher yields associated with early planting have been reported [24,37,40,41,42,43,44,45]. Early planting allows for a longer growing season, which includes longer vegetative and reproductive growth phases, resulting in larger yields [46]. Moreover, the shortened duration of vegetative growth and seed filling period associated with delayed planting contributes to low yields [36,47]. However, higher yields in late planting, as is the case in WMAFSF in this study, have been reported in cowpeas [42]. Under the conditions of this study, the technique of delaying planting may not be effective for managing bean leaf beetle populations and foliar damage. Because a larger yield is the ultimate goal of crop production [43], early planting may be more appropriate, especially for the two agro-ecological zones of NMF and WNF.

The effect of spray regimes on bean leaf beetle abundance was only distinct between spray regime 6 and the control. However, for foliar damage, the more the sprays, the less the foliar damage, with spray regime 6 recording significantly lower foliar damage than the other spray regimes across the three planting times. Additionally, higher marketable grain yield was recorded from spray regime six while the lowest was recorded from the control across all planting times. Insecticides have been reported to reduce insect pests damage and increase yield [25,26,27,28,29,30,48]. Our study suggests that insecticides, especially cypermethrin and imidacloprid, are effective and can be used in the management of bean leaf beetles. However, the higher costs associated with the insecticides coupled with the low value of the bean crop means that only two sprays are economical. This is evidenced by the resultant marginal rate of returns that is less than one in spray regimes (except spray regime 5 in WNF and WMAFSF). The marginal rate of returns associated with spray regime 2, 3, and 4 in NMF is due to higher yields recorded in NMF than the other two agro-ecological zones. Only spray regime 5 had a marginal rate of returns that is greater than one in all the three agro-ecological zones, suggesting that only two sprays are economical. Future studies should be conducted to determine the timing of the two sprays in order to benefit from beans. Farmers will benefit from fewer insecticide applications in terms of reduced costs and pesticide exposure, as well as less environmental effects [31]. In addition, future research on the economic thresholds and economic injury levels of bean leaf beetles will inform better management of this pest complex in Uganda. Similar research in the future should look further into the effects of the two seasons. Furthermore, the best practice for each region from this manuscript should be tested further with other integrated pest management tactics.

## 5. Conclusions

Application of insecticides reduced foliar damage and increased grain yield with a combination of soil drench and foliar application resulting in the highest grain yield. However, this did not result in economic benefits. Early planting resulted in the lowest bean leaf beetle abundance and foliar damage in two of the three agro-ecological zones. Similarly, early planting resulted in the highest marketable grain yield in all agro-ecological zones, suggesting that delayed planting may not be beneficial to farmers. Furthermore, grain yields were significantly increased when insecticide spray regimes were combined with early planting in all agro-ecological zones during both seasons. However, application of insecticides should be based on accurate economic thresholds, which need to be established for bean leaf beetles. 

## Figures and Tables

**Table 1:** Planting dates for the three planting times in different agro-ecological zones.

Season	Agro-Ecological Zone	District	Planting Time	Planting Date
2016	NMF	Lira	Early planting	18 July 2016
			Mid planting	2 August 2016
			Late planting	10 September 2016
	WNF	Arua	Early planting	12 July 2016
			Mid planting	2 August 2016
			Late planting	15 August 2016
	WMAFSF	Hoima	Early planting	25 August 2016
			Mid planting	10 September 2016
			Late planting	7 October 2016
2017	NMF	Lira	Early planting	8 April 2017
			Mid planting	23 April 2017
			Late planting	23 May 2017
		Gulu	Early planting	7 April 2017
			Mid planting	27 April 2017
			Late planting	25 May 2017
	WNF	Arua	Early planting	24 April 2017
			Mid planting	26 May 2017
			Late planting	24 June2017
	WMAFSF	Hoima	Early planting	4 April 2017
			Mid planting	20 April 2017
			Late planting	23 May 2017

**Table 2:** Interaction effects of season, agro-ecological zone and planting time on bean leaf beetle abundance.

Season	Agro-Ecological Zone	Planting Time		
		Early Planting	Mid Planting	Late Planting
2016	NMF	0.710 ± 0.003 ^a^	0.709 ± 0.003 ^a^	0.710 ± 0.003 ^a^
	WNF	0.713 ± 0.003 ^ab^	0.736 ± 0.003 ^d^	0.713 ± 0.003 ^ab^
	WMAFSF	0.711 ± 0.003 ^a^	0.709 ± 0.003 ^a^	0.709 ± 0.003 ^a^
2017	NMF	0.738 ± 0.003 ^de^	0.747 ± 0.003 ^ef^	0.722 ± 0.003 ^bc^
	WNF	0.729 ± 0.003 ^cd^	0.748 ± 0.003 ^f^	0.716 ± 0.003 ^ab^
	WMAFSF	0.709 ± 0.003 ^a^	0.708 ± 0.003 ^a^	0.708 ± 0.003 ^a^

Values are means ± standard errors. Values within a row bearing the same letters are not significantly different at α = 0.05.

**Table 3:** Interaction effects of season, agro-ecological zone, and planting time on foliar damage (per plant).

Season	Agro-Ecological Zone	Planting Time		
		Early Planting	Mid Planting	Late Planting
2016	NMF	0.75 ± 0.11 ^bc^	0.92 ± 0.11 ^cde^	0.92 ± 0.11 ^cde^
	WNF	0.79 ± 0.11 ^bc^	1.16 ± 0.11 ^fg^	0.99 ± 0.11 ^de^
	WMAFSF	1.33 ± 0.11 ^h^	1.06 ± 0.11 ^ef^	1.55 ± 0.11 ^i^
2017	NMF	1.09 ± 0.11 ^ef^	1.05 ± 0.11 ^ef^	1.28 ± 0.11 ^gh^
	WNF	0.75 ± 0.11 ^b^	1.08 ± 0.11 ^ef^	0.88 ± 0.11 ^bcd^
	WMAFSF	0.53 ± 0.11 ^a^	0.79 ± 0.11 ^bc^	0.86 ± 0.11 ^bcd^

Values are means ± standard errors. Values within a row bearing the same letters are not significantly different at α = 0.05.

**Table 4:** Interaction effects of season, agro-ecological zone and spray regimes on foliar damage (per plant).

Season	Agro-Ecological Zone	Spray Regimes						
		Spray Regime 1	Spray Regime 2	Spray Regime 3	Spray Regime 4	Spray Regime 5	Spray Regime 6	Control
2016	NMF	0.66 ± 0.10 ^def^	0.88 ± 0.10 ^fghijkl^	1.04 ± 0.10 ^ijklmno^	1.08 ± 0.10 ^klmnop^	1.12 ± 0.10 ^nopq^	0.10 ± 0.10 ^a^	1.18 ± 0.10 ^mnopqr^
	WNF	0.90 ± 0.10 ^ghijkl^	0.98 ± 0.10 ^ijklmno^	1.10 ± 0.10 ^klmnop^	1.21 ± 0.10 ^opqr^	1.20 ± 0.10 ^nopqr^	0.12 ± 0.10 ^a^	1.36 ± 0.10 ^qrs^
	WMAFSF	0.86 ± 0.10 ^fghijk^	1.18 ± 0.10 ^mnopqr^	1.68 ± 0.10 ^tu^	1.65 ± 0.10 ^tu^	1.68 ± 0.10 ^tu^	0.36 ± 0.10 ^bc^	1.81 ± 0.10 ^u^
2017	NMF	0.73 ± 0.10 ^efgh^	0.99 ± 0.10 ^hijklmno^	1.28 ± 0.10 ^pqr^	1.38 ± 0.10 ^rs^	1.53 ± 0.10 ^st^	0.48 ± 0.10 ^cd^	1.60 ± 0.10 ^stu^
	WNF	0.72 ± 0.10 ^defg^	0.84 ± 0.10 ^fghij^	0.97 ± 0.10 ^hijklmn^	1.08 ± 0.10 ^klmnop^	1.12 ± 0.10 ^lmnopq^	0.40 ± 0.10 ^bc^	1.20 ± 0.10 ^nopqr^
	WMAFSF	0.50 ± 0.10 ^cde^	0.65 ± 0.10 ^def^	0.80 ± 0.10 ^fghi^	0.90 ± 0.10 ^ghijkl^	0.94 ± 0.10 ^ghijklm^	0.23 ± 0.10 ^ab^	1.06 ± 0.10 ^jklmnop^

Values are means ± standard errors. Values within a row bearing the same letters are not significantly different at α = 0.05.

**Table 5:** Interaction effects of season, planting time and spray regimes on foliar damage (per plant).

Season	Planting Time	Spray Regimes						
		Spray Regime 1	Spray Regime 2	Spray Regime 3	Spray Regime 4	Spray Regime 5	Spray Regime 6	Control
2016	Early planting	0.62 ± 0.11 ^cd^	0.89 ± 0.11 ^efgh^	1.19 ± 0.11 ^ijklmno^	1.23 ± 0.11 ^jklmno^	1.27 ± 0.11 ^klmnop^	0.21 ± 0.11 ^a^	1.31 ± 0.11 ^mnopq^
	Mid planting	0.84 ± 0.11 ^defg^	1.02 ± 0.11 ^ghijk^	1.28 ± 0.11 ^lmnop^	1.30 ± 0.11 ^mnopq^	1.32 ± 0.11 ^nopq^	0.09 ± 0.11 ^a^	1.50 ± 0.11 ^pq^
	Late planting	0.96 ± 0.11 ^fghi^	1.12 ± 0.11 ^hijklmn^	1.35 ± 0.11 ^nopq^	1.42 ± 0.11 ^opq^	1.42 ± 0.11 ^opq^	0.27 ± 0.11 ^a^	1.55 ± 0.11 ^qs^
2017	Early planting	0.55 ± 0.11 ^bc^	0.70 ± 0.11 ^cde^	0.90 ± 0.11 ^efgh^	0.98 ± 0.11 ^ghij^	1.02 ± 0.11 ^ghijk^	0.31 ± 0.11 ^ab^	1.06 ± 0.11 ^ghijklm^
	Mid planting	0.69 ± 0.11 ^cde^	0.89 ± 0.11 ^efgh^	1.04 ± 0.11 ^ghijkl^	1.17 ± 0.11 ^ijklmno^	1.23 ± 0.11 ^jklmno^	0.54 ± 0.11 ^bc^	1.29 ± 0.11 ^lmnop^
	Late planting	0.70 ± 0.11 ^cdef^	0.89 ± 0.11 ^efgh^	1.11 ± 0.11 ^hijklmn^	1.22 ± 0.11 ^jklmno^	1.36 ± 0.11 ^nopq^	0.26 ± 0.11 ^a^	1.50 ± 0.11 ^pq^

Values are means ± standard errors. Values within a row bearing the same letters are not significantly different at α = 0.05.

**Table 6:** Interaction effects of season, agro-ecological zone, and planting time on marketable grain yield (Kgs/Ha).

Season	Agro-Ecological Zone	Planting Time		
		Early Planting	Mid Planting	Late Planting
2016	NMF	921 ± 103 ^i^	334 ± 103 ^bc^	140 ± 103 ^a^
	WNF	820 ± 103 ^gh^	449 ± 103 ^def^	412 ± 103 ^cde^
	WMAFSF	890 ± 103 ^hi^	463 ± 103 ^def^	503 ± 103 ^ef^
2017	NMF	928 ± 103 ^i^	1154 ± 103 ^j^	1307 ± 103 ^k^
	WNF	749 ± 103 ^g^	728 ± 103 ^g^	406 ± 103 ^cd^
	WMAFSF	443 ± 103 ^def^	286 ± 103 ^b^	509 ± 103 ^f^

Values are means ± standard errors. Values within a row bearing the same letters are not significantly different at α = 0.05.

**Table 7:** Interaction effects between season, agro-ecological zone, and spray regimes on marketable grain yield (Kgs/Ha).

Season	Agro-Ecological zone	Spray Regimes						
		Spray Regime 1	Spray Regime 2	Spray Regime 3	Spray Regime 4	Spray Regime 5	Spray Regime 6	Control
2016	NMF	508 ± 106 ^defghij^	474 ± 106 ^cdefghi^	424 ± 106 ^bcdefg^	419 ± 106 ^bcdefg^	390 ± 106 ^bcdef^	656 ± 106 ^jkl^	386 ± 106 ^bcdef^
	WNF	620 ± 106 ^hijk^	643 ± 106 ^ijkl^	527 ± 106 ^efghij^	492 ± 106 ^defghij^	487 ± 106 ^defghij^	811 ± 106 ^lmn^	342 ± 106 ^abcd^
	WMAFSF	631 ± 106 ^hijk^	611 ± 106 ^hijk^	612 ± 106 ^hijk^	547 ± 106 ^fghijk^	521 ± 106 ^efghij^	928 ± 106 ^no^	480 ± 106 ^cdefghi^
2017	NMF	1207 ± 106 ^p^	1182 ± 106 ^p^	1005 ± 106 ^o^	995 ± 106 ^o^	1002 ± 106 ^o^	1526 ± 106 ^q^	990 ± 106 ^o^
	WNF	713 ± 106 ^klm^	624 ± 106 ^hijk^	579 ± 106 ^ghijk^	549 ± 106 ^fghijk^	465 ± 106 ^cdefgh^	933 ± 106 ^no^	531 ± 106 ^fghij^
	WMAFSF	519 ± 106 ^efghij^	392 ± 106 ^bcedf^	354 ± 106 ^abcde^	308 ± 106 ^abc^	274 ± 106 ^ab^	853 ± 106 ^mno^	188 ± 106 ^a^

Values are means ± standard errors. Values within a row bearing the same letters are not significantly different at α = 0.05.

**Table 8:** Interaction effects of season, planting time, and spray regimes on marketable grain yield (Kgs/Ha).

Season	Planting Time	Spray Regimes						
		Spray Regime 1	Spray Regime 2	Spray Regime 3	Spray Regime 4	Spray Regime 5	Spray Regime 6	Control
2016	1	906 ± 107 ^nopqr^	950 ± 107 ^opqr^	898 ± 107 ^nopq^	819 ± 107 ^lmnop^	777 ± 107 ^ijklmno^	1087 ± 107 ^r^	702 ± 107 ^ghijklm^
	2	461 ± 107 ^bcdef^	379 ± 107 ^abc^	347 ± 107 ^ab^	354 ± 107 ^ab^	355 ± 107 ^ab^	726 ± 107 ^ghijklmn^	286 ± 107 ^ab^
	3	392 ± 107 ^abcd^	399 ± 107 ^abcde^	317 ± 107 ^ab^	285 ± 107 ^ab^	266 ± 107 ^a^	582 ± 107 ^efgh^	220 ± 107 ^a^
2017	1	812 ± 107 ^klmnop^	668 ± 107 ^ghijklm^	605 ± 107 ^fghi^	634 ± 107 ^fghijk^	620 ± 107 ^fghij^	1025 ± 107 ^qr^	580 ± 107 ^efgh^
	2	794 ± 107 ^jklmno^	796 ± 107 ^jklmno^	756 ± 107 ^hijklmn^	571 ± 107 ^defg^	570 ± 107 ^defg^	994 ± 107 ^pqr^	578 ± 107 ^efgh^
	3	833 ± 107 ^mnop^	733 ± 107 ^ghijklmn^	647 ± 107 ^defgh^	647 ± 107 ^ghijkl^	552 ± 107 ^cdefg^	1294 ± 107 ^s^	551 ± 107 ^cdefg^

Notes: Planting time; 1 = Early planting, 2 = Mid planting, 3 = Late planting. Values are means ± standard errors. Values within a row bearing the same letters are not significantly different at α = 0.05.

**Table 9:** The relationship between foliar damage and marketable grain yield.

Season	Agro-Ecological Zone	Site	Planting Time	Intercept	Slope	R2	*p*-Value
2016	NMF	On farm	Early planting	1012.6	−45.7	0.0079	0.251
			Mid planting	429.8	−136.9	0.2874	*p* < 0.001
			Late planting	270.0	−129.8	0.2693	*p* < 0.001
		On station	Early planting	952.2	−116.3	0.0534	*p* < 0.05
			Mid planting	674.8	−335.9	0.4986	*p* < 0.001
			Late planting	229.0	−110.6	0.2907	*p* < 0.001
	WNF	On farm	Early planting	1018.8	−262.1	0.1604	*p* < 0.001
			Mid planting	348.6	13.5	0.0025	0.516
			Late planting	478.1	−91.5	0.1170	*p* < 0.001
		On station	Early planting	973.8	−194.7	0.1631	*p* < 0.001
			Mid planting	892.1	−305.9	0.3352	*p* < 0.001
			Late planting	611.0	−181.0	0.2188	*p* < 0.001
	WMAFSF	On farm	Early planting	1292.7	−93.4	0.0328	*p* < 0.05
			Mid planting	1000.2	−142.7	0.0610	*p* < 0.05
			Late planting	589.8	−119.3	0.3493	*p* < 0.001
		On station	Early planting	836.7	−144.3	0.2170	*p* < 0.001
			Mid planting	182.4	−96.1	0.2970	*p* < 0.001
			Late planting	836.7	−144.3	0.2170	*p* < 0.001
2017	NMF	On farm	Early planting	1338.6	−134.4	0.0154	0.109
			Mid planting	1847.6	−235.3	0.0380	*p* < 0.05
			Late planting	2165.6	−427.9	0.3218	*p* < 0.001
		On station	Early planting	650.2	−2.1	0.00002	0.952
			Early planting	885.5	−156.2	0.1406	*p* < 0.001
			Late planting	1160.5	−146.1	0.1914	*p* < 0.001
	WNF	On farm	Early planting	889.1	−140.3	0.0394	*p* < 0.05
			id planting	1287.7	−132.1	0.0471	*p* < 0.05
			Late planting	846.1	−94.6	0.0536	p<0.05
		On station	Early planting	884.9	−203.2	0.2155	*p* < 0.001
			Mid planting	353.2	−38.2	0.0116	0.164
			Late planting	84.1	−40.0	0.1099	*p* < 0.001
	WMAFSF	On farm	Early planting	295.3	−109.7	0.0765	*p* < 0.001
			Mid planting	354.9	−282.7	0.2741	*p* < 0.001
			Late planting	1033.6	−587.8	0.2150	*p* < 0.001
		On station	Early planting	831.4	−285.2	0.2360	*p* < 0.001
			Mid planting	397.0	−72.9	0.1075	*p* < 0.001
			Late planting	757.9	−404.4	0.5206	*p* < 0.001

## Data Availability

The data presented in this study are available upon request from the corresponding author.

## Supplementary Materials

The following supporting information can be downloaded at: https://www.mdpi.com/article/10.3390/insects13080709/s1, Table S1: Summary of Rainfall, Temperature and Relative humidity data from the three agro-ecological zones during 2016 and 2017; Table S2: Anova table of factors analyzed in the model for bean leaf beetle abundance; Table S3: Anova table of factors analyzed in the model for foliar damage; Table S4: Anova table of factors analyzed in the model for marketable grain yield; Table S5: Mean marketable grain yield, gross and net returns, cost of sprays and marginal rate of returns for spray regimes in different agro-ecological zones.
