# Supplementary material for: Bean Leaf Beetle (Ootheca spp.) (Coleoptera: Chrysomelidae) Management via Planting Timing and Insecticides
Source: Insects. 2022 Aug 7;13(8):709. doi: 10.3390/insects13080709 (PMC9408959; doi:10.3390/insects13080709)
Supplement: Supplementary file 1 [file insects-13-00709-s001.zip › insects-1783736-supplementary.pdf]

Table S1. Summary of rainfall, temperature and relative humidity from the three agro-ecological zones during 2016 and 2017 [5]

**Season**	**Month**	**Northern Moist Farmlands**					**West Nile Farmlands**					**Western Mid-Altitude Farmlands and the Semliki Flats**				
		Temp (°C)		RH (%)		RF (mm)	Temp (°C)		RH (%)		RF (mm)	Temp (°C)		RH (%)		R/F (mm)
		Max	Min	9am	3pm		Max	Min	9am	3pm		Max	Min	9am	3pm	
2016	Jul	28.6	18.4	86	57	204.3	26.9	17.1	86	62	173.7	28.4	15.7	85	57	71.1
	Aug	29.4	18.3	81	55	72.7	27.5	16.7	84	58	71.1	28.7	17.1	83	59	87.8
	Sep	28.1	19.6	76	52	119.6	28.1	16.6	83	58	171.5	27.8	17.7	83	60	142.7
	Oct	28.0	18.2	73	48	143.4	28	17	78	57	155.9	28.2	18.1	79	58	290.8
	Nov	28.7	18.7	72	46	59.0	28.7	17.3	75	52	98.4	29.0	18.3	78	56	101.6
	Dec	30.6	18.8	59	29	0	30.6	16.9	63	38	0.0	30.9	17.7	65	38	34.9
	Mean	28.9	18.7	74.8	47.8	99.9	28.3	16.9	78.2	54.2	111.8	28.8	17.4	78.8	54.7	121.5
2017	Jan	35.0	18.4	45	20	3.3	32.7	17.3	59	25	1.1	33.5	17.1	61	26	6.1
	Feb	34.5	18.7	61	31	34.6	32.2	18.3	61	35	14.3	32.5	18.4	70	38	92.8
	Mar	31.9	20.7	78	47	181.8	32.1	18.8	70	39	99.7	31.3	18.7	73	46	112.5
	Apr	31.8	21.0	79	42	151.9	30	18.4	75	50	129.4	30.6	19.0	75	52	113.3
	May	29.5	20.7	84	57	138.1	28.8	18	79	55	128.0	29.4	18.7	82	56	130.6
	Jun	30.2	20.6	80	53	166.1	28.3	17.4	82	66	146.4	29.2	18.1	78	59	253.1
	Mean	32.2	20.0	71.2	41.6	112.6	30.7	18.0	71.0	43.3	86.5	31.1	18.3	73.2	46.2	118.1

Temp=temperature, RF=rainfall, RH=relative humidity

Table S2. Anova table of factors analyzed in the model for bean leaf beetle abundance

**Effect**	**Sum sq**	**Mean Sq**	**df**	**F Value**	**Pr (>F)**
Season (S)	0.209	0.209	1	190.246	p<0.001
Agro-ecological zone (AZ)	0.332	0.166	2	151.173	p<0.001
Planting time (P)	0.179	0.090	2	81.599	p<0.001
Spray regime (SP)	0.021	0.004	6	3.152	P<0.05
S × AZ	0.187	0.093	2	84.943	p<0.001
S × P	0.037	0.019	2	16.873	p<0.001
AZ × P	0.151	0.038	4	34.403	p<0.001
S × SP	0.006	0.001	6	0.852	0.529
AZ×SP	0.010	0.001	12	0.736	0.717
P × SP	0.004	0.000	12	0.288	0.991
S ×AZ× P	0.033	0.008	4	7.430	p<0.001
S × AZ × SP	0.014	0.001	12	1.046	0.403
S × P × SP	0.010	0.001	12	0.725	0.728
AZ × P × SP	0.008	0.000	24	0.288	0.999
S × AZ × P × SP	0.019	0.001	24	0.719	0.838

Notes: df = Degrees of freedom

Table S3. Anova table of factors analyzed in the model for foliar damage

**Effect**	**Sum Sq**	**Mean sq**	**Df**	**F Value**	**Pr (>F)**
Season (S)	25.90	25.90	1	103.81	p<0.001
Agro-ecological zone (AZ)	7.19	3.59	2	14.40	p<0.001
Planting time (P)	44.09	22.04	2	88.36	p<0.001
Spray regime (SP)	751.54	125.26	6	502.07	p<0.001
S × AZ	190.90	95.45	2	382.59	p<0.001
S × P	2.70	1.35	2	5.42	P<0.05
AZ × P	42.94	10.74	4	43.03	p<0.001
S × SP	25.71	4.29	6	17.18	p<0.001
AZ×SP	13.71	1.14	12	4.58	p<0.001
P × SP	9.39	0.78	12	3.14	p<0.001
S ×AZ× P	28.60	7.15	4	28.66	p<0.001
S × AZ × SP	25.42	2.12	12	8.50	p<0.001
S × P × SP	10.94	0.91	12	3.66	p<0.001
AZ × P × SP	19.31	0.81	24	3.22	p<0.001
S × AZ × P × SP	18.77	0.78	24	3.14	p<0.001

Notes: df = Degrees of freedom

Table S4. Anova table of factors analyzed in the model for Marketable grain yield

**Effect**	**Sum sq**	**Mean sq**	**D.F**	**F Value**	**Pr (>F)**
Season (S)	46492201	46492201	1	489.72	p<0.001
Agro-ecological zone (AZ)	85306325	42653163	2	449.28	p<0.001
Planting time (P)	74161156	37080578	2	390.58	p<0.001
Spray regime (SP)	128532929	21422155	6	225.65	p<0.001
S × AZ	199768106	99884053	2	1052.11	p<0.001
S × P	92133510	46066755	2	485.23	p<0.001
AZ × P	18387809	4596952	4	48.42	p<0.001
S × SP	6106574	1017762	6	10.72	p<0.001
AZ×SP	3356660	279722	12	2.95	p<0.001
P × SP	4290578	357548	12	3.77	p<0.001
S ×AZ× P	56171492	14042873	4	147.92	p<0.001
S × AZ × SP	3981858	331821	12	3.50	p<0.001
S × P × SP	10562335	880195	12	9.27	p<0.001
AZ × P × SP	15658283	652428	24	6.87	p<0.001
S × AZ × P × SP	14460537	602522	24	6.35	p<0.001

Notes: df = Degrees of freedom

Tables S5. Mean marketable grain yield, gross and net returns, cost of sprays and marginal rate of returns for treatments in different agro-ecological zones.

Agro-ecological zone	Treatments	Cost of chemical (USD. Ha^{-1})	Cost of application (USD. Ha^{-1})	Total costs (USD)	Marketable yield (Kgs. Ha^{-1})	Gross returns (USD. Ha^{-1})	Net returns (USD. Ha^{-1})	Marginal returns
NMF	Spray regime 1	284.3	94.6	378.9	848.7	619.6	240.6	0.6
	Spray regime 2	237.1	80.0	317.1	869.7	634.9	317.8	1.0
	Spray regime 3	192.9	65.4	258.3	735.3	536.8	278.5	1.1
	Spray regime 4	146.3	50.8	197.1	698.8	510.1	313.0	1.6
	Spray regime 5	99.3	36.2	135.5	712.5	520.1	384.6	2.8
	Spray regime 6	1220.0	193.6	1413.6	1046.5	763.9	-649.7	-0.5
	Control				685.8	500.6	500.6	
WNF	Spray regime 1	294.6	94.6	389.2	657.3	479.8	90.6	0.2
	Spray regime 2	245.4	80.0	325.4	700.2	511.1	185.7	0.6
	Spray regime 3	193.8	65.4	259.2	573.9	418.9	159.8	0.6
	Spray regime 4	143.9	50.8	194.7	519.4	379.2	184.5	0.9
	Spray regime 5	96.7	36.2	132.9	454.5	331.8	198.9	1.5
	Spray regime 6	1230.3	193.6	1423.9	879.9	642.3	-781.6	-0.5
	Control				419.4	306.2	306.2	
WMAFSF	Spray regime 1	310.3	94.6	404.9	661.9	483.2	78.3	0.2
	Spray regime 2	265.4	80.0	345.4	634.6	463.3	117.8	0.3
	Spray regime 3	218.0	65.4	283.4	641.3	468.1	184.7	0.7
	Spray regime 4	164.1	50.8	214.9	536.6	391.7	176.8	0.8
	Spray regime 5	107.6	36.2	143.8	570	416.1	272.3	1.9
	Spray regime 6	1245.9	193.6	1439.5	939.3	685.7	-753.8	-0.5
	Control				499.4	364.6	364.6	

NMF=Northern Moist Farmlands, WNF=West Nile Farmlands, WMAFSF= Western Mid-Altitude Farmlands and the Semliki Flats. Values are averaged over planting times, sites and season
